# Visual Genome-Wide RNAi Screening to Identify Human Host Factors Required for *Trypanosoma cruzi* Infection

**DOI:** 10.1371/journal.pone.0019733

**Published:** 2011-05-20

**Authors:** Auguste Genovesio, Miriam A. Giardini, Yong-Jun Kwon, Fernando de Macedo Dossin, Seo Yeon Choi, Nam Youl Kim, Hi Chul Kim, Sung Yong Jung, Sergio Schenkman, Igor C. Almeida, Neil Emans, Lucio H. Freitas-Junior

**Affiliations:** 1 Image Mining Group, Institut Pasteur Korea, Seongnam-si, Gyeonggi-do, South Korea; 2 Center for Neglected Diseases Drug Discovery, Institut Pasteur Korea, Seongnam-si, Gyeonggi-do, South Korea; 3 Discovery Biology Group, Institut Pasteur Korea, Seongnam-si, Gyeonggi-do, South Korea; 4 Department of Biochemistry and National Research Laboratory, Yonsei University, Seoul, South Korea; 5 Departamento de Microbiologia, Imunologia e Parasitologia, Universidade Federal de São Paulo, São Paulo, São Paulo, Brazil; 6 Department of Biological Sciences, The Border Biomedical Research Center, University of Texas at El Paso, El Paso, Texas, United States of America; 7 High Throughput Biology Group, Synthetic Biology ERA, CSIR Biosciences, Pretoria, South Africa; Federal University of São Paulo, Brazil

## Abstract

The protozoan parasite *Trypanosoma cruzi* is the etiologic agent of Chagas disease, a neglected tropical infection that affects millions of people in the Americas. Current chemotherapy relies on only two drugs that have limited efficacy and considerable side effects. Therefore, the development of new and more effective drugs is of paramount importance. Although some host cellular factors that play a role in *T. cruzi* infection have been uncovered, the molecular requirements for intracellular parasite growth and persistence are still not well understood. To further study these host-parasite interactions and identify human host factors required for *T. cruzi* infection, we performed a genome-wide RNAi screen using cellular microarrays of a printed siRNA library that spanned the whole human genome. The screening was reproduced 6 times and a customized algorithm was used to select as hits those genes whose silencing visually impaired parasite infection. The 162 strongest hits were subjected to a secondary screening and subsequently validated in two different cell lines. Among the fourteen hits confirmed, we recognized some cellular membrane proteins that might function as cell receptors for parasite entry and others that may be related to calcium release triggered by parasites during cell invasion. In addition, two of the hits are related to the TGF-beta signaling pathway, whose inhibition is already known to diminish levels of *T. cruzi* infection. This study represents a significant step toward unveiling the key molecular requirements for host cell invasion and revealing new potential targets for antiparasitic therapy.

## Introduction

Chagas disease, also known as American trypanosomiasis, is a neglected disease that affects millions of people in the Americas. The Drugs for Neglected Diseases *initiative* [DND*i*] (http://www.dndi.org/diseases/chagas/global-view.html) estimates that more than 8 million people are infected and 100 million people are at risk of infection [1]. Chagas disease accounts for 667,000 disability-adjusted life years (DALYs) in Latin America and kills more people than any other parasite-borne disease, making it the most important parasitic disease on the continent [2].

Previously confined to endemic countries in Latin America, increasing numbers of patients are being reported in non-endemic developed countries such as the United States, Spain, Australia and Japan due to an increased influx of people from endemic countries unknowingly carrying the parasite [3].

Despite being significantly debilitating and causing great social and economic disruption, Chagas disease is still considered a disease of poverty. Current chemotherapy has proven effective for the acute phase of the disease that is recognized only in an estimated 1–2% of all individuals acquiring the infection [4]. Therefore, there is no efficient drug for the vast majority of patients who are in the chronic phase of the disease. The current treatment options, Nifurtimox and Benznidazole, show low efficacy and numerous side effects (http://www.dndi.org/diseases/chagas/current-treatment.html) [5]. Although new drugs could potentially solve the problem, Chagas disease is one of the most neglected diseases in terms of drug development [6].

*Trypanosoma cruzi* is the etiologic agent of Chagas disease, and it progresses through four developmental stages during its life cycle, alternating between insect vectors and mammalian hosts [7]. This protozoan parasite is able to invade and multiply inside a broad range of mammalian cells. Different routes of invasion mediated by distinct cell surface receptors, secondary messengers, and transcription factors have been described [8], [9]. Once inside the host cells, *T. cruzi* trypomastigotes disrupt the parasitophorous vacuole to escape to the cytosol and immediately transform into amastigotes. These forms multiply by binary fission, differentiating back into trypomastigotes before bursting out of the host cell to invade surrounding cells or reach the bloodstream to infect remote tissues [7].

*T. cruzi* isolates present extensive biological, biochemical and genetic diversity [10]–[12]. The clinical manifestations of the disease can vary from a symptomless infection to a severe chronic disease with cardiovascular or gastrointestinal involvement. Genetic variation of both the host and the parasite likely plays key roles in the outcome of the disease, suggesting genetic individuality of parasite clones [13], [14]. At least 6 different subgroups of *T. cruzi* have recently been recognized based on genetic, molecular or immunological markers [12]. Equally elaborated is the multi-step process used by these parasites to enter and multiply into their host cells, involving various parasitic and cellular molecules and ultimately leading to intracellular calcium mobilization in both cells and parasites [9], [15], [16]. Taken together, these studies highlight the complexity of this parasite and of its interaction with the host and thus explain why the molecular requirements for parasite intracellular growth and persistence are not yet fully understood.

The use of reverse genetic tools such as RNA interference (RNAi) therefore represents an alternative strategy to identify host proteins that might be important for *T. cruzi* invasion, intracellular parasite survival and proliferation. Recently, it has been shown that silencing laminin γ-1 expression in cultured human coronary artery smooth muscle cells rendered them significantly more resistant to parasite attachment and intracellular proliferation [17]. Using a similar approach, the same authors demonstrated that stable interference of thrombospondin-1 expression in cultured HeLa cells *in vitro* resulted in an increased resistance to *T. cruzi* invasion [18]. Moreover, silencing cytokeratin 18 inhibited intracellular proliferation of Y and CL strains of *T. cruzi* in HeLa cells [19]. Other experiments, including transcriptome profiling and host gene-expression analyses of *T. cruzi*-infected cells [20]–[22], have uncovered many possible key players in this interaction; however, only some of the host metabolic and signaling pathways were found to be shared by different cell types [20].

Recently, several genome-wide RNAi screens have been used to elucidate the mechanisms involved in host-pathogen interactions [23]–[25], all of them using microplate-based assays. In addition, several research groups have recently published studies identifying the host proteins required for HIV infection using similar microplate-based approaches [26]–[28], although surprisingly very little overlap was found among the identified proteins. This discrepancy could be explained, according to the authors, by small technical differences between the experiments [28] that may have been further evidenced by the variations between microplate wells. In order to achieve a more homogeneous screening, we used a new approach relying on the use of glass microarrays that permitted us to increase the throughput and eliminate well-to-well variations, generating an unbiased and more uniform analysis.

A prototype of a cellular microarray-based RNAi screening over glass slides method was first described by Erfle and collaborators in 2004 [29] and was further developed for high-throughput scale in genome-wide screens investigating mitosis, cell cycle progression and constitutive protein secretion machinery [30]–[32]. This type of reverse transfection approach shows several advantages over microplate-based screens: i) reduced costs due to the small amount of small interfering RNA [siRNA] and other reagents needed, ii) faster data acquisition due to the high number of experiments per array, and iii) low heterogeneity due to the absence of physical barriers between experiments, thus increasing screening data quality [30].

In this study, we used a cellular microarray-based RNAi screening as a primary step to search for human cell factors that play a role during infection by the protozoan parasite *T. cruzi*. The strongest primary screening hits were subsequently submitted to a secondary screening and later confirmed using individual siRNAs in two different cell lines. Overall, our screening strategy allowed us to identify and validate 14 genes whose silencing impaired *T. cruzi* infection, providing clues about the molecular mechanisms that guide the infection process.

## Methods

### Chemicals

All chemicals were purchased from Sigma-Aldrich. DRAQ5 was purchased from BioStatus (Shepshed, UK). All siRNA duplexes were purchased from Dharmacon (USA). The siRNA library comprised 0.5 nM of the Dharmacon siARRAY whole human genome siRNA library (Thermofisher, West Lafayette, CO) containing 84,508 siRNAs corresponding to four unique siRNA duplexes, targeting 21,127 unique human genes. Primary antibody against p65 was purchased from Santa Cruz Biotechnology (Santa Cruz, CA) and the fluorescent secondary antibody Alexa Fluor 488 was purchased from Molecular Probes, Invitrogen (Carlsbad, CA). Transfection reagents were purchased from Qiagen (Valencia, CA). All culture media and their supplements were purchased from Gibco (Invitrogen, Carlsbad, CA).

### Microarray Printing

siRNA transfection solution was prepared as described previously [29], [30], [32]–[34] and printed as 3888 spot arrays (108×36 spots) on No. 1 glass coverslips (Marienfeld, Germany) using stealth pins (Telechem, USA) and a high-throughput microarray printer (Genomic Solutions, USA) at 22–25°C, 55–65% RH enclosed in a custom built clean chamber providing a sterile HEPA filtered atmosphere. To facilitate spot localization, siGLO Red dye (Dharmacon, Thermofisher) was also incorporated into the transfection solution. Printed arrays were stored in a desiccating chamber and showed no significant alterations in performance from one week up to 21 months post-printing. Seven printed slides covered a single human genome in siRNA and contained 2% of control siRNA spots (scrambled siRNA).

### Cell and Parasite Cultures

U2OS, HeLa and LLC-MK2 cells (ATCC, Manassas, VA) were cultured in DMEM high-glucose medium supplemented with 10% fetal bovine serum (FBS) and 1% penicillin/streptomycin in a humid atmosphere of 5% CO_2_ at 37°C.

U2OS are osteosarcoma cells that can be easily transfected with siRNAs and infected by *T. cruzi* parasites, being successfully adapted to our microarray model. They present a large cytoplasm that facilitates parasite detection and grow preferentially in monolayers, which is crucial for individual cell quantification during software analysis. Little overlap has been observed among different infected host cells in mRNA profiling studies [20], so to check if our hits were cell-specific, we also tested the individual siRNAs in HeLa cells, a cell line more commonly used for *T. cruzi* infection.studies.

LLC-MK2 (monkey kidney epithelial cells) were used to maintain *in vitro* culture of trypomastigotes. Tissue culture-derived trypomastigotes (TCTs) of *Trypanosoma cruzi* strain Dm28c [35] were harvested from the supernatants of infected LLC-MK2 cultures maintained in DMEM Low Glucose medium (Gibco) containing 2% FBS and 1% penicillin/streptomycin. *T. cruzi* Dm28c strain belongs to the subgroup or discrete typing unit I (DTU TcI) based on the most recent nomenclature [12].

### Validation of the Microarray Screening and p65 Immunofluorescent Detection

To validate the transfection efficiency of the microarray screen, 2×10^6^ U2OS cells were seeded onto reverse transfection arrays comprising 250–300 µm spots spaced at 500 µm intervals on a glass wafer (class 1, 130–160 µm thickness). Spots contained either p65 specific or control siRNAs in addition to a red fluorescent oligonucleotide (siGLO Red) in an encapsulation mixture and were printed using SMP9 pins in a stealth printhead mounted in a Gene Machines Omnigrid HT array spotter. Cells were incubated for 48 hours at 37°C to allow for silencing.

For the immunofluorescent detection of p65, cells were washed twice with phosphate-buffered saline (PBS), fixed for 10 minutes with 4% (w/v) paraformaldehyde in PBS, and then washed once with PBS. Permeabilization was performed using 0.1% Triton X-100 in PBS for 15 min, followed by washing in PBS. All the procedures above were performed at room temperature. Cells were then incubated with a 1∶200 dilution of rabbit anti–p65 antibody in 10% goat serum overnight at 4°C. On the following day, cells were washed 3 times with PBS for 10 minutes on an orbital rotator and incubated with Alexa Fluor 488 goat anti-rabbit secondary antibody (1∶800) for 60 minutes at room temperature. Cells were washed 3 times for 10 minutes each with PBS on an orbital shaker prior to the addition of 5 µM of DRAQ5 in PBS and incubation for 10 minutes at room temperature. Confocal images were acquired using an ImageXpress Ultra point scanning confocal microscope (Molecular Devices, USA). Quantification of p65 silencing was performed using MetaXpress software (Molecular Devices), dividing the average p65 intensity by total cell number of each acquired image.

### Primary Screening and Microarray Infection

For the Primary Screening, U2OS cells were plated at a density of 1×10^6^ cells/dish onto printed siRNA arrays and cultivated in Opti-MEM I medium supplemented with 5% FBS and 1% penicillin/streptomycin in a humid atmosphere of 5% CO_2_ at 37°C for 48 hours. Cells were infected with 1×10^6^ TCTs/ml in 25 ml (approximate ratio of 25 parasites per cell) and incubated for 8 hours at 37°C. After that period, free TCTs were washed out and cells were cultivated in fresh Opti-MEM I supplemented medium at 37°C for additional 28 hours. At a total of 36 hours after the beginning of the infection, arrays were fixed in 4% (w/v) paraformaldehyde in Dulbecco's phosphate-buffered saline (DPBS, Invitrogen) at room temperature and stained with 2.5 µM DRAQ5 before imaging (see Figure S1).

### Microarray Acquisition

Images covering the entire surface of the slides were acquired with a 20× objective using an ImageXpress Ultra microscope and directly saved as 16 bit TIFF files on an external database. Each scanned slide comprised 1820 confocal pictures in two channels (siGLO Red and DRAQ5) with a size of 1000×1000 16-bit pixels each. Consequently, the size of an acquired array was roughly 7 gigabytes, and hence a genome screen composed of 7 arrays was roughly 50 gigabytes total. The genome screening was replicated 6 times. Because each array contained 3888 spots, we obtained a total of 163,296 visual experiments and acquired 76,440 images. Images were read directly from the database for image reconstruction and analysis using software designed for this purpose (described below).

### Software Development for Spot Recognition

We developed in-house dedicated software, called IM, which allowed for the assemblage, miniaturization, grid fitting, image reconstruction and analysis of each individual spot. Each spotted experiment could lie either on one or across two to four pictures because the pictures were acquired regardless of spot location. A miniature image of the whole slide was produced by reducing the size of 1820 images by a factor of 3/100. Adaptive grid fitting was applied to identify siRNA spots on the miniature image of the array. Each spot had coordinates that reported them to the high resolution image database that was used to extract pieces of pictures needed to reconstruct individual annotated spotted experiments.

### Software Development for Image Analysis

Dedicated image analysis was developed and integrated into the same software to quantify individual cells in each experiment. Briefly, an algorithm was designed to optically address each spot where the siRNAs were printed and another was created to artificially emphasize nuclei and parasites in different colors. The resulting artificial two-color images were used by the software to detect and quantify each individual cell and parasites per cell. For the primary screening, we retrieved cell number and the median number of parasites per cell over a spot. For secondary and tertiary screenings, we retrieved the ratio of infected cells (infected was defined as a cell containing at least one parasite), the median and the average number of parasites per cell, and the average number of parasites per infected cell.

### Data Analysis during Primary Screening

There were 6 spotted experiments (replicates) per gene in the primary screening. Cell distribution was normalized in each array prior to analysis and the number of cells and median number of parasites per cell were calculated for each visual experiment. We then selected the replicates that simultaneously presented a cell number higher than the mean minus two standard deviations and a median number of parasites lower than the mean minus two standard deviations when compared to the negative control distribution (scrambled siRNA). Cell number was considered along with the number of parasites per cell in order to remove from the analysis genes that when knocked down were toxic to the cells. The selection based on these two criteria represented the results of roughly 3.75% of all experiments. Hits were extracted from this selection using the method described below.

If a set of 6 experiments were to be selected randomly among all experiments, then 3.75% would fall into the selected experiments. Therefore, we compared the ratio of experiments falling in the selection for 6 experiments (replicate) of each gene to the ratio for a random sample. Following this concept, a gene was defined as a hit when the ratio of its selected replicates was at least five times higher than the ratio of a randomly chosen experiment. For example, for the gene MGC33951, the exact results were as follows: 2 of its 6 replicate siRNA spots were accidentally not printed, two fell into the selected area using the criteria described above and two fell outside. Therefore, 50% (2 among 4) of the MGC33951 replicates fell into 3.75% of the total selected experiments, showing a ratio score of 0.07 = 0.5/0.0375 that is lower than 0.2 (0.2 corresponds to a ratio 5 times higher 5 = 1/0.02). The score of 0.07 is also much lower than 1, which corresponds to a random selection. A score of 0.07 represents a probability 14 times higher for a replicate of that gene to fall in the selection (equivalent to a p-value of 1.84×10E-4) and therefore cannot be explained just by chance. Using this method, a total of 277 genes were selected.

### Validation of Microplate Format and Secondary Screening

To confirm the hits identified during primary screening, we performed a secondary screen in 96-well plates. To validate this format, U2OS cells were transfected with either scrambled or p65 specific siRNA, as suggested by the manufacturer and subsequently infected with *T. cruzi* trypomastigotes and immunostained for p65 protein. Briefly, U2OS cells were trypsinized one day prior to transfection, diluted in fresh DMEM high glucose medium supplemented with 5% FBS without antibiotics and transferred to 96-well plates (Greiner). A total of 5000 U2OS cells were seeded per well and cultured for 16 hours. Transient transfection of siRNAs was performed using DharmaFECT 1 (Dharmacon, Thermofisher). For each well, 9.9 µl of serum-free DMEM and 0.1 µl of DharmaFECT 1 were preincubated for 5 minutes at room temperature. At the same time, 5 µl of serum-free DMEM was mixed with 5 µl of each siRNA (1 µM) and incubated for 5 minutes at room temperature. The two mixtures were combined and incubated for 20 minutes at room temperature to allow for complex formation. After the addition of 80 µl of complete DMEM medium to the mixture, the entire solution was added to the cells in each well, resulting in a final concentration of 50 nM for the siRNAs. After transfection, cells were incubated for 48 hours to allow gene silencing. Cells were infected with 1×10^6^ parasites/ml (100 µl/well) for 8 hours before free parasites were washed out. Cells and intracellular parasites were then incubated in fresh DMEM supplemented medium for additional 28 hours. Cells and parasites were fixed and stained with DRAQ5 as described for the primary screening. In addition, cells were immunostained for p65 protein under the following conditions: in the absence of parasites, in the absence of siRNA, in the presence of scrambled siRNA or in the presence of p65 siRNA. Labeling intensity was measured using the MetaXpress software (Molecular Devices).

After visual inspection, 162 hits selected from the primary screening hit list were assayed again in a secondary screening. All of the procedures for seeding, transfection, infection and staining were exactly the same as described for the validation assay. The secondary screening experiment was performed in duplicate.

To further select the hits from the secondary screening and assess more precisely how the parasite infection was modified by the knockdown of genes, four parameters were considered during the analysis as described above: i) ratio of infected cells, ii) median parasite number per cell, iii) average number of parasites per cell, and iv) number of parasites per infected cell. The selected genes were those ones that scored lower than the mean minus one time the standard deviation of the negative control distribution (scrambled siRNA) in at least one of the four parameters mentioned above, in both duplicates. The genes that came out only in one of the replicates were discarded. Using this method, a total of 15 genes were selected.

### Confirming Specificity of the siRNAs

To exclude possible off-target effects due to pooled siRNA duplexes, a third assay using single siRNA duplexes was performed for 13 of the hits identified in the secondary screen. For these experiments, all procedures were performed in exactly the same way described for the secondary screen, except that 4 single siRNA duplexes were individually tested to silence each gene. The four parameters assessed to confirm the hits were the same as those described for the secondary screen (see above). The selected genes were those that scored lower than the mean minus one time the standard deviation of the negative control distribution (scrambled siRNA) in at least one of the four parameters in both duplicates. All the hit genes tested were confirmed using this method.

The methodology used for this validation was similar to the one performed with U2OS. The experiment was repeated twice and three parameters were taken into consideration when selecting hits: i) ratio of infected cells, ii) average number of parasites per cell, and iii) number of parasites per infected cell. The hit genes were those ones that scored lower than the mean minus one time the standard deviation of the negative control (scrambled siRNA) in at least one of the parameters mentioned above in both duplicates. Eleven hits were also confirmed in HeLa cells.

### RNA Extraction

Total RNA was extracted from U2OS (up to 10^6^) cells using Trizol reagent (Invitrogen). Target RNA was reverse transcribed using the MMLV reverse transcriptase enzyme (Promega). In the first step, 5 µM Oligo(dT)_16_ was added to 0.5–1 µg of total RNA and annealed at 70°C for 10 min. Then, 100 U MMLV reverse transcriptase was added in the presence of 50 mM Tris-HCl, pH 8.3, 75 mM KCl, 3 mM MgCl_2_, and 5 mM unlabeled deoxynucleotides (dNTPs) and incubated at 37°C for 60 min. For each experiment, RT-minus controls were included to provide a negative control for subsequent PCR reactions. To minimize variations in reverse transcriptase efficiency, all samples from a single experiment were reverse transcribed simultaneously.

### Real-Time PCR

Real-time PCR was performed using SYBR Premix Ex Taq II (TaKaRa Bio Inc.) and a MJ Research PTC-200 Thermo Cycler (BioRad). Template cDNAs were diluted ten-fold and 1–5 µl were used for amplification in a 20 µl final volume containing 10 µl of SYBR Premix Ex Taq II and 0.4 µM of each primer (Table S1). The protocol included an initial denaturation step at 95°C for 15 min, followed by 32 cycles of 95°C for 20 sec, 60–65°C for 30 sec, and 72°C for 30 sec. After amplification, a melting curve was obtained by increasing temperatures from 65°C to 95°C with fluorescence detection at 0.2°C intervals.

The quantification of target gene expression was performed using the cycle threshold (Ct) value in a PCR amplification curve by cluster analysis with variable cluster endpoints. Data were determined from duplicate assays. For normalization, the cell number in the specimen was determined from the GAPDH gene quantification.

## Results

### Microarray Validation

We proved the concept of our microarray methodology by validating two different controls. The first control was p65 protein, a component of the NF-κB complex, primarily chosen because its knockdown could be easily assessed by immunofluorescence staining. The other control was scrambled siRNA, a siRNA sequence that does not target any gene in the human genome and should not affect the knockdown of p65 protein to any extent when transfected into cells.

**Figure 1:**
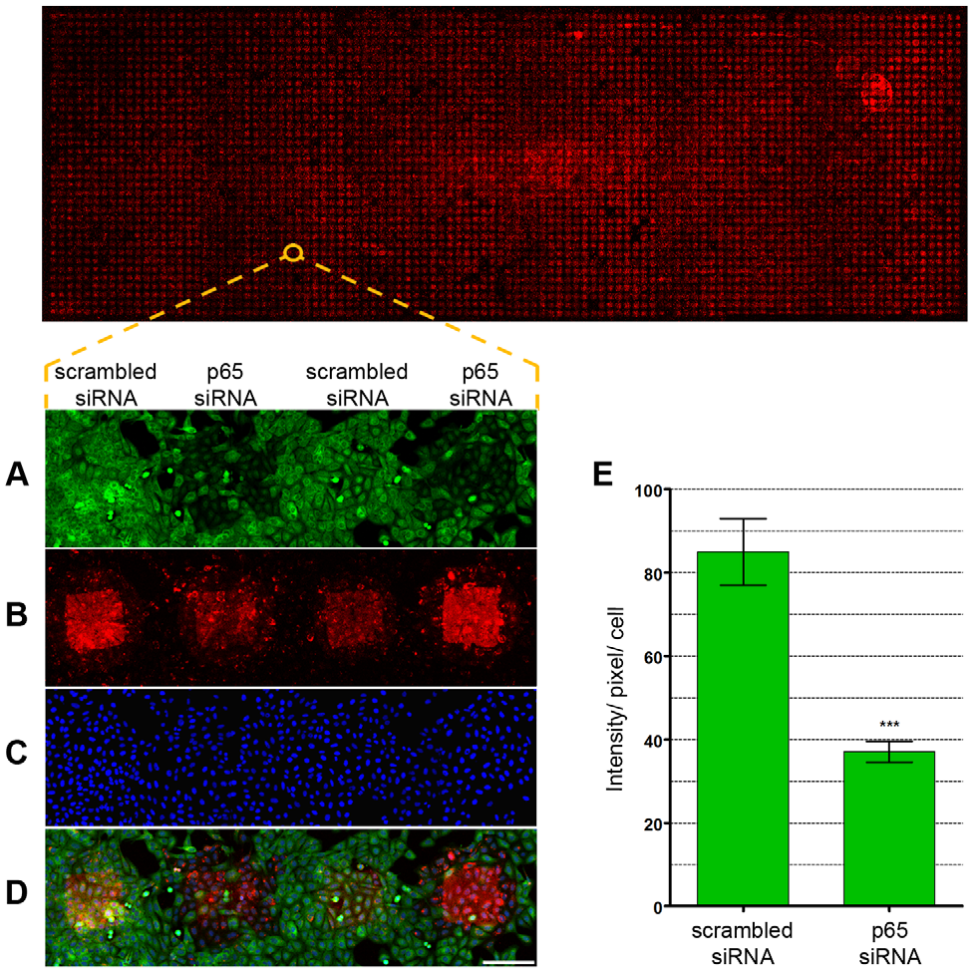
Proof-of-concept of the microarray screening. U2OS cells were seeded onto reverse transfection arrays comprising 250–300 µm spots spaced center-to-center at 500 µm intervals on a glass wafer. Spots contained p65 specific or control siRNAs (scrambled siRNA) and a red fluorescent siRNA in an encapsulation mixture (siGLO Red). Cells were incubated for 48 hours and then fixed and stained with anti-p65 antibodies. Confocal images were acquired 48 hours post-transfection. Scrambled siRNA was chosen as a negative control because it does not target any gene in the human genome. (**A**) Anti-p65 labeling of two p65 and two scrambled siRNA spots. (**B**) Spot images labeled with siGLO Red. (**C**) DNA staining (DRAQ5) showing an equal distribution of cells. (**D**) Overlay image. Scale bar represents 250 µm. (**E**) Quantification of p65 silencing using MetaXpress software (Molecular Devices). Plot showing p65 labeling (intensity/pixel/cell) for cells outside spots or overlaid on scrambled siRNA or p65 siRNA containing spots. ***p<0.0001 (unpaired t-test), n = 8.

For this validation, cells were transfected with either p65 or scrambled siRNA; after silencing, the cells were fixed and immunostained against p65 protein. Images were acquired in three channels, one showing the p65 immunofluorescence (Figure 1A), a second highlighting the spots where the siRNAs were printed (Figure 1B) and a third showing the cell nuclei (Figure 1C). When we analyzed the overlay image of the 3 channels (Figure 1D), it was clear that knockdown of p65 was successfully achieved and was siRNA-specific because its expression was decreased only in the cells overlaid on p65 siRNA-containing spots. The p65 labeling intensity of cells transfected with the p65 siRNA was significantly reduced (56.4% lower, p<0.0001, n = 8) in comparison to cells transfected with scrambled siRNA (Figure 1E), further confirming knockdown under the primary screening assay conditions.

### Software Development for Image Analysis

**Figure 2:**
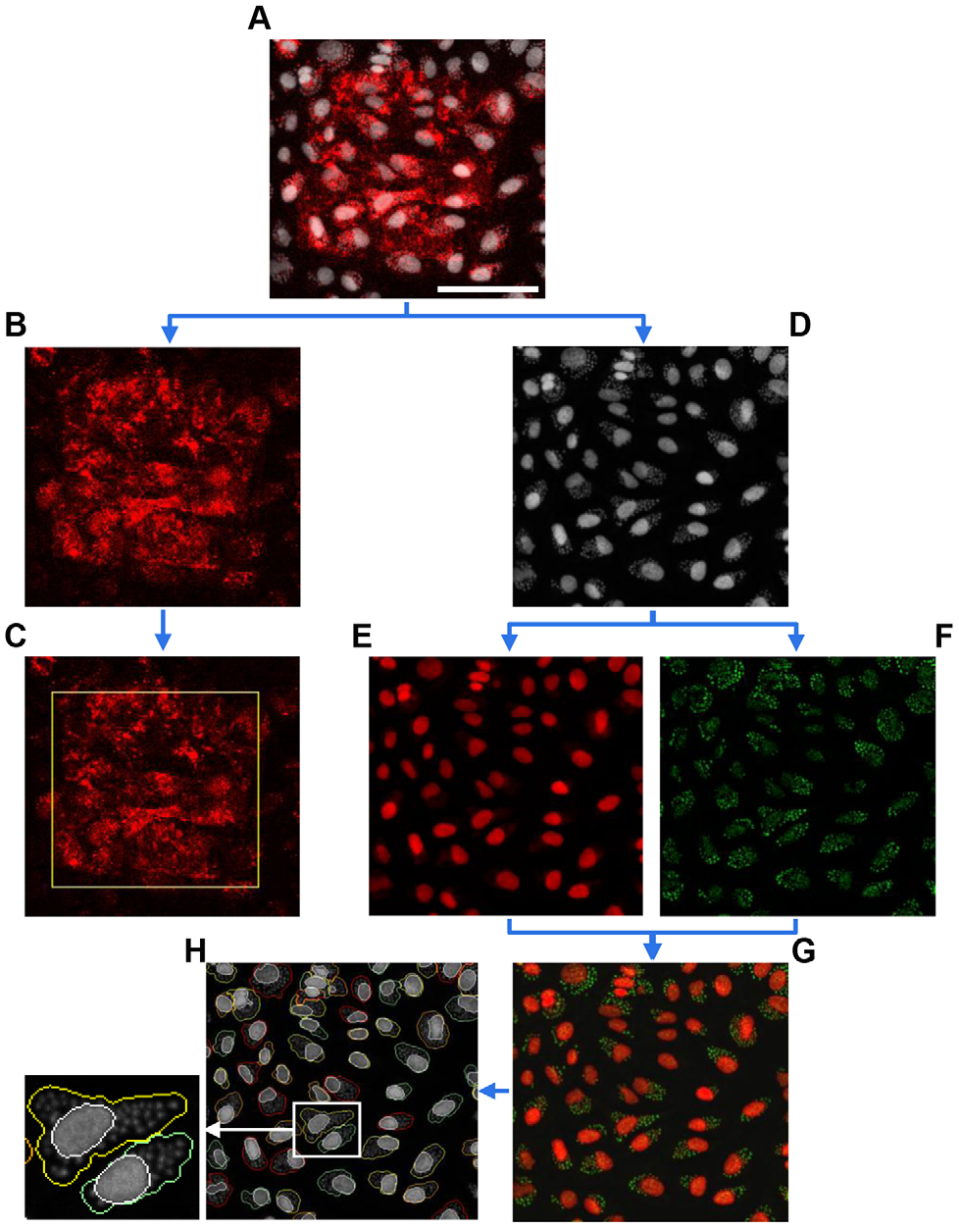
Microarray screening image analysis. Schematic illustration of how the developed software recognized the siRNA spot, the cells and the parasites for subsequent measurements. (**A**) All images of the *T. cruzi* primary screening were acquired in two different channels, as shown in (**B**) and (**D**). (**B** and **C**) Optically addressable siRNA spots were labeled with siGLO Red to enable their localization on the arrays. (**D**) Cell and parasite nuclei were stained using DRAQ5. As the raw images were acquired in a single wavelength, cells and parasites were artificially separated and emphasized in different colors (**E** and **F**, respectively), generating artificial two-wavelength images (**G**). This allowed for individual cell detection and quantification of several independent descriptors to measure infection (**H**). An enlarged panel showing cell detection by the software in greater detail is in the bottom left. The cell nuclei and boundaries are outlined in white and yellow/green, respectively (arbitrary colors). Scale bar represents 150 µm.

Dedicated image analysis software was developed to interpret the data obtained during the screenings. The original images of the primary screen, for example, were acquired in two channels (Figure 2A): one channel shows a spot stained with siGLO Red (Figure 2B), and another shows cell and parasite nuclei stained with DRAQ5 (Figure 2D). An algorithm was designed to optically address each spot (Figure 2C) and hence facilitate gene localization. In addition, there was a need to distinguish cell nuclei from parasite nuclei stained with DRAQ5 because they were both acquired in the same wavelength channel (Figure 2D). A second dedicated algorithm employed a sequence of morphological operators and region growing to artificially emphasize nuclei in one color (Figure 2E) and parasites in another color (Figure 2F). This generated artificial two-color image (Figure 2G) could be easily used by the software to detect each cell and parasite individually and therefore quantify cells and parasites per cell (Figure 2H). A real time movie of spot detection and analysis during the primary screen can be seen in the Supporting Information section (Video S1).

### Primary Screening Using Microarrays

The genome-wide screening was performed in 6 independent experiments to obtain a good statistical sampling and to ensure that no spot would be missed. If each spot is defined as representing a single experiment, then our data corresponded to a total of 163,296 experiments.

We found some host genes that, when knocked down, impaired trypomastigote entrance and/or amastigote proliferation inside host cells. They accounted for 277 genes that received different scores based on their performance in terms of percentage of infected cells, median parasite number per cell, and number of parasites per cell.

**Figure 3:**
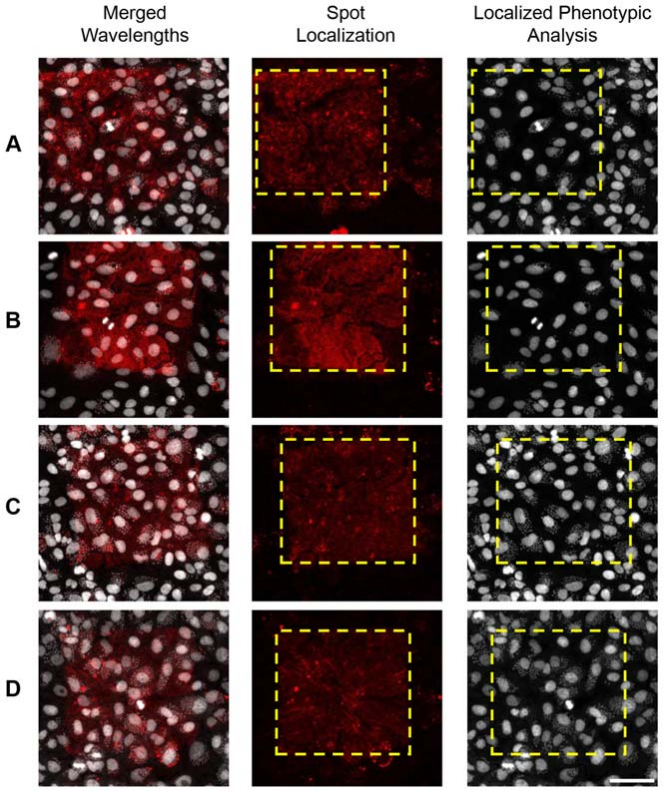
Genome-wide microarray screening results. Examples of data from the primary screening performed over microarrays showing that the knockdown of some genes inhibited infection by *T. cruzi* parasites. In (**A**) and (**B**), two examples are shown of genes selected as hits: PCDHA13 (protocadherin alpha 13) and C20orf200 (chromosome 20 open reading frame 200), respectively. Scrambled siRNA (**C**) was used as a negative control and p65 siRNA (**D**) was used as a transfection control (see Materials and Methods). Cells and parasites are pseudocolored in white, while siRNA spots are pseudocolored in red. The yellow dashed boxes outline the spot region. Cells within the spot were silenced for the indicated genes and compared with the cells lying outside the spot for their infection ratio and parasite load through software analysis. Scale bar represents 80 µm.

Two examples of these genes are shown in Figures 3A and 3B. The gene coding for protocadherin alpha 13 [PCDHA13] (Figure 3A) was found among the first 126 hits and had a p-value of 8.72E-04. The chromosome 20 open reading frame 200 [C20orf200] (Figure 3B) scored among the first 85 hits, showing a p-value of 4.48E-04. As a negative control for the experiment, approximately 2% of all the microarray spots contained scrambled siRNAs (Figure 3C). Moreover, specific siRNAs targeting p65 protein, as used in our proof-of-concept experiment shown in Figure 1, were also spotted on the microarrays to ensure transfection efficiency, and they showed no effect over *T. cruzi* infection (Figure 3D).

### Secondary Screening and Confirmation of siRNAs Specificity

**Figure 4:**
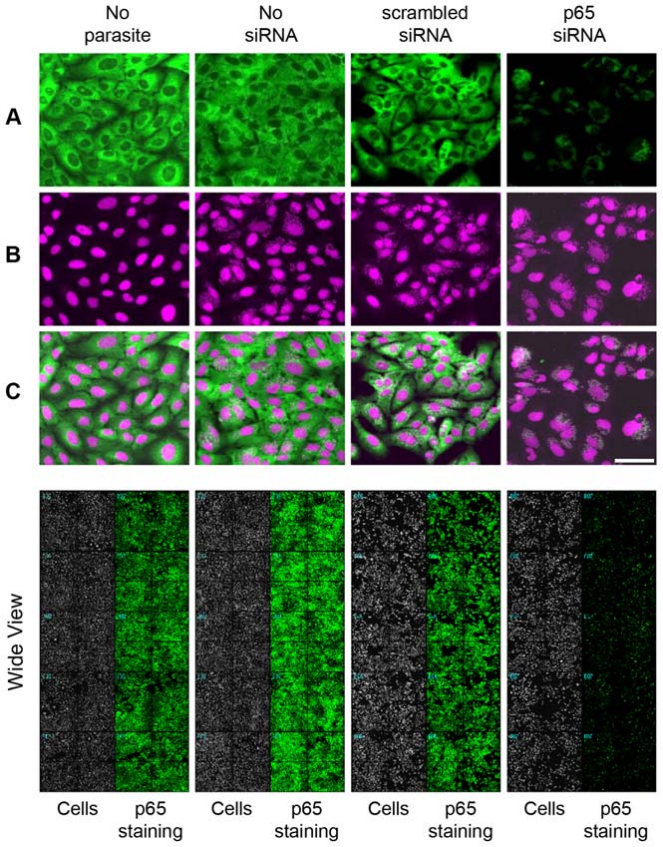
Validation of the secondary screening. The secondary screening was performed in 96-well plates. To validate this format, U2OS cells were seeded, transfected with the appropriate siRNAs and then infected with *T. cruzi* trypomastigotes as detailed in Materials and Methods. From left to right, cells were immunostained for p65 protein in the absence of *T. cruzi* parasites, in the absence of siRNAs, in the presence of scrambled siRNA or in the presence of p65 siRNA, respectively. (**A**) Immunofluorescence staining against p65 protein (green). (**B**) DNA staining of cell and parasite nuclei (DRAQ5, purple). (**C**) Overlay images. In the bottom panel, a wide view of the plate wells, showing p65 protein labeling (green) and cells (white). The rightmost column evidences the p65 knockdown when cells were transfected with p65 siRNA. Scale bar represents 80 µm.

The 162 genes with the highest scores were retested in a secondary screening assay in 96-well plates that is a robust and well-established format regularly used for screenings. As was performed for the microarray technology, this 96-well format also had to be validated before proceeding with the screening. Therefore, U2OS cells were transfected with scrambled siRNA or p65 siRNA and subsequently immunostained for p65 protein. The amount of p65 protein present in the cytoplasm of cells transfected with p65-targeting siRNA (Figure 4A, rightmost column) was lower than the amount found in scrambled siRNA-transfected cells (Figure 4A, second column from right to left). Moreover, this lower amount of cytoplasmic protein was revealed to be independent of parasite or siRNA presence (Figure 4A, two leftmost columns). Nuclei staining is shown in Figure 4B and overlay images in Figure 4C. The overlay images, as well as the wide view of the experiment plate in the bottom panel, clearly demonstrate the silencing of the p65 protein (rightmost column) in comparison to other treatments.

After validating the plate format, a secondary screening was carried out in duplicates containing the strongest 162 hits selected previously from the primary screening. Fifteen genes were confirmed as hits because their silencing reproduced the inhibitory effect on the *T. cruzi* infection process. However, one of the hits (LOC400729) was not considered for further analysis because its record was discontinued from Entrez Gene public database. The remaining 14 human genes that proved to be important for *T. cruzi* infection are listed on Table 1.

**Table 1:** List of the 14 human genes that are important for *T. cruzi* infection.

Gene Symbol*	Gene Name*	GeneID*	UniProtKB/Swiss-Prot#	Subcellular Location#	Molecular Function#	Biological Process#
CHP	Calcium binding protein P22	11261	Q99653	Cytoplasm	Calcium ion binding	Potassium ion transport
					Potassium channel regulator activity	Small GTPase mediated signal transduction
CABP2	Calcium binding protein 2	51475	Q9NPB3	Cytoplasm > perinuclear region	Calcium ion binding	Signal transduction
				Cell membrane > Lipid-anchor > Cytoplasmic side		
				Golgi apparatus		
CCL4L1	Chemokine (C-C motif) ligand 4-like 1	9560	Q8NHW4	Secreted	Chemokine activity	Chemotaxis
						Immune response
						Inflammatory response
CDH11	Cadherin 11, type 2, OB-cadherin (osteoblast)	1009	P55287	Cell membrane	Calcium ion binding	Homophilic cell adhesion
				Single-pass type I membrane protein	Protein binding	Ossification
C20orf200	Chromosome 20 open reading frame 200	253868	Q96NR2	Unknown	Unknown	Unknown
FLJ32783	RIB43A domain with coiled-coils 1	158787	Q8N443	Unknown	Unknown	Unknown
FUT8	Fucosyltransferase 8 (alpha (1,6) fucosyltransferase)	2530	Q9BYC5	Golgi apparatus > Golgi stack membrane	SH3 domain binding	L-fucose catabolic process
				Single-pass type II membrane protein	Glycoprotein 6-alpha-L-fucosyltransferase activity	N-glycan processing
						In utero embryonic development
						Oligosaccharide biosynthetic process
						Protein amino acid glycosylation in Golgi
LOC131873	Collagen, type VI, alpha 6	131873	A6NMZ7	Secreted > extracellular space > extracellular matrix (Note: Deposed in the extracellular matrix of skeletal muscle)	Protein binding	Cell adhesion
LOC389895	Hypothetical LOC389895	389895	-	Unknown	Unknown	Unknown
LOC401993	Olfactory receptor, family 2, subfamily T, member 5	401993	Q6IEZ7	Cell membrane	Olfactory receptor activity	GPCR protein signaling pathway
				Multi-pass membrane protein		Response to stimulus
						Sensory perception of smell
MGC33951	Chromosome 15 open reading frame 43	145645	Q8NHR7	Unknown	Unknown	Unknown
NICE-3	Chromosome 1 open reading frame 43	25912	Q9BWL3	Membrane	Unknown	Unknown
				Single-pass membrane protein		
PCDHA13	Protocadherin alpha 13	56136	Q9Y5I0	Cell membrane	Calcium ion binding	Homophilic cell adhesion
				Single-pass type I membrane protein	Protein binding	
PRIMA1	Proline rich membrane anchor 1	145270	Q86XR5	Cell membrane	Unknown	Neurotransmitter catabolic process
				Single-pass type I membrane protein		
				Cell junction		
				Cell junction > synapse		

*Data from Entrez Gene (http://www.ncbi.nlm.nih.gov/gene/).

# Data from UniProtKB (http://www.uniprot.org/uniprot/).

**Figure 5:**
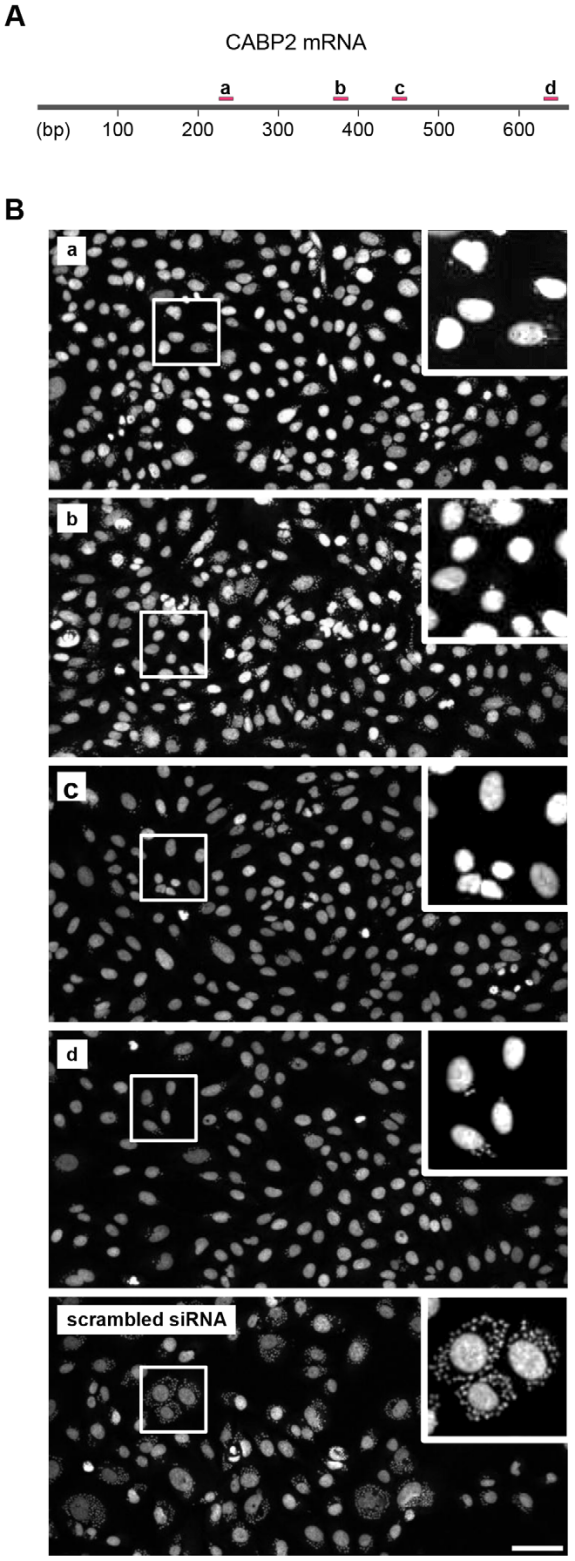
Confirming specificity of siRNA silencing. To confirm specificity and exclude off-target effects of the siRNA pools used in the primary and secondary screens, the 4 different siRNA duplexes from the pool targeting each gene were separated and individually transfected to U2OS cells in 96-well plates following the same experimental conditions used for secondary screen. (**A**) Schematic representation of the four siRNAs targeting the calcium binding protein 2 (CABP2) mRNA sequence. bp = base pairs. (**B**) Pictures showing CABP2 gene silencing using the four different siRNAs depicted in (**A**). U2OS cells were transfected with each one of the siRNAs and then infected with *T. cruzi* parasites. Pictures **a, b, c** and **d** show a decrease in infection when compared to scrambled siRNA (bottom picture). All 13 genes tested were confirmed by at least two individual siRNAs, demonstrating that the infection inhibition seen in primary and secondary screens was not due to an off target effect. Scale bar represents 80 µm.

In addition, we performed two new assays as a means to confirm the specificity of the siRNAs and exclude possible off-target effects caused by the siRNA pools used during the primary and secondary screenings. For the first assay, the four individual siRNA duplexes that composed the pool for each gene were separated and individually transfected into U2OS cells in 96-well plates following the same experimental conditions used for secondary screening. Because individual siRNA duplexes targeting the olfactory receptor family 2 subfamily T member 5 [LOC401993] were not available for purchase at the time these experiments were conducted, this validation assay was performed with the remaining 13 genes (see Table 1). All genes tested were confirmed as hits by at least two of the siRNA duplexes (data not shown), suggesting that the siRNA pools used previously were specific to their corresponding genes and that the inhibitory effect that occurred upon infection was not due to off-target effects. The four individual siRNA duplexes silencing calcium binding protein 2 [CABP2], for example, target different regions of the gene and do not overlap each other (Figure 5A). In this case, even when transfected individually, each of the four siRNA duplexes was able to silence the CABP2 gene in host cells, resulting in a reduced infection by *T. cruzi* parasites in comparison to scrambled siRNA-transfected cells (Figure 5B).

In a second validation assay, we decided to check if these hits were cell-specific, since a previous study showed that little overlap is found in the signaling pathways affected by *T. cruzi* infection in different cell lines [20]. With this aim, we performed the same experiment with individual siRNAs using HeLa cells instead of U2OS cells, because HeLa is commonly used as the host cell in *T. cruzi* invasion assays [9]. Most of the genes validated with individual siRNAs in U2OS cells also interfered with infection of HeLa cells, except LOC389895 (Hypothetical LOC389895) and MGC33951 (Chromosome 15 open reading frame 43) that were found to be specific for the osteosarcoma cell line (data not shown).

**Figure 6:**
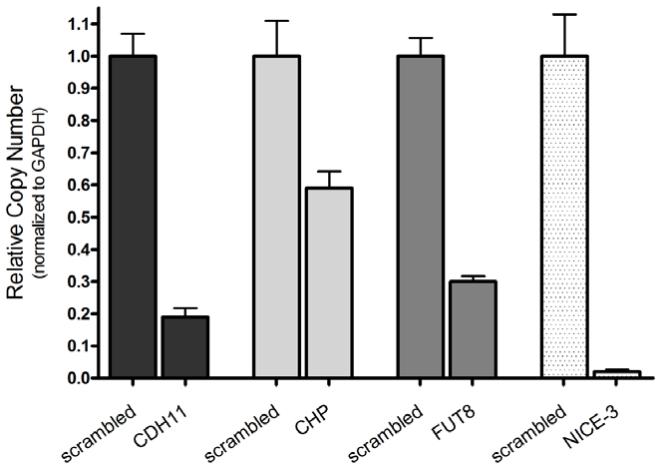
Checking mRNA levels by quantitative real-time PCR. U2OS cells were transfected with the siRNAs (scrambled siRNA, CDH11, CHP, FUT8 and NICE-3) and total RNA was isolated from each sample after a 48-hour incubation. cDNAs were prepared and endogenous mRNA levels were measured using real-time PCR. The relative copy numbers of the transcripts were normalized to GAPDH and the knockdown was quantified using scrambled siRNA as the negative control.

To evaluate the messenger RNA [mRNA] transcript levels of the genes after transfection with the siRNAs, we selected four hits and performed quantitative real-time polymerase chain reaction [qRT-PCR] assays using the SYBR Green method. The quantification of the target genes was performed using the cycle threshold [Ct] value in a PCR amplification curve. The results showed that the transcripts of the 4 genes were down regulated when compared to scrambled siRNA-transfected samples, knocking down approximately 80% of cadherin 11 type 2 OB-cadherin [CDH11], 40% of calcium binding protein P22 [CHP], 70% of fucosyltransferase 8 [FUT8], and more than 95% of chromosome 1 open reading frame 43 [NICE-3] mRNA transcripts (Figure 6).

## Discussion

*T. cruzi* is able to invade almost any nucleated mammalian cell type and facilitates its own entry by actively manipulating the host cell [8], [9]. This complex interaction between *T. cruzi* and its host cells during the process of infection is primarily mediated by interplay of signaling cascades that involve both parasitic and cellular factors [36]. Some of the parasites' primary tools for entering host cells during the infection process are their surface glycoproteins, in particular mucins and trans-sialidases, and their specific proteinase cruzipain [37]. Although several important parasite factors have been discovered, little is known about the roles of host cell proteins in this process. The limited information available reveals that some cell receptors, including cytokeratin 18, galectin-3 and P74, may be involved in parasite adhesion to mammalian cells [19], [38], [39]. Recent evidence also indicates that *T. cruzi* is able to modulate the extracellular matrix network to promote its invasion of human cells, increasing levels of laminin γ-1 and thrombospondin-1, and binding to fibronectin [17], [18], [40].

RNA interference (RNAi) has increasingly been used to uncover the roles of specific host cell proteins, demonstrating the effects of individual gene silencing on *T. cruzi* infection [17]–[19]. However, these experiments were usually focused on genes belonging to pathways previously identified as important for the *T. cruzi* infection process. To broaden the view of the host genes that are affected by *T. cruzi* infection, Costales and colleagues have recently studied the transcriptional response triggered by *T. cruzi* infection in phenotypically diverse human cell types [20]. They found that only a small fraction of host metabolic and signaling pathways were shared by the different cell types tested, suggesting that intrinsic host cell metabolic differences might be determining factors for the response to infection. Imai and colleagues [21] also performed a microarray analysis of host gene expression during the intracellular nest formation of *T. cruzi* amastigotes. However, his results showed little or no overlap with Costales' results, indicating that each single condition used during the experiment, i.e., cell type, parasite strain or time of infection, could have dramatic effects on the output of the assay.

The aim of this work was to better understand the interactions between *T. cruzi* and its host cells by searching for cell factors that could affect the *T. cruzi* infective process. Our first step was to develop a genome-wide RNAi screen to be as unbiased and homogeneous as possible, eliminating the physical separations present in microplate-based assays and taking advantage of the great number of experiments performed simultaneously in a single array. This primary screening using the microarray methodology served as a first filter to provide us with a smaller number of hits, and it can be used successfully in the future to understand other mechanisms and host-parasite interactions in different organisms.

In a secondary screening, we confirmed the inhibitory effects of 15 genes on the infection ratio among 162 genes that showed a strong inhibitory effect. The fact that only about 10% of the hits were confirmed in the secondary screening must be a consequence of subtle differences that can affect *T. cruzi* infection. In the primary screening, the intricate combination of a sensitive microarray format with this complex parasite, and the lack of a positive control as a direct phenotype during analysis, led us to use simple parameters to extract the hits and therefore increase the number of artifacts. By contrast, the microplate format used for the secondary screening likely offered higher assay stringency in comparison with the microarray format and consequently restricted the number of hits obtained. This higher stringency could be explained by factors such as more homogeneous distribution of cells and different transfection conditions. Nevertheless, for our purposes, the priority was to confirm that the hits were real and robust, and therefore we decided to further explore these results.

Screenings that use siRNA pools have several significant advantages over identical screens using corresponding individual siRNA duplexes, especially for high-throughput purposes. However, despite generating more evident phenotypes than any of the corresponding single siRNA duplexes, these pools might result in some false positive hits [41]. To evaluate the veracity of the hits from the secondary screening, we tested the knockdown of 13 genes using 4 separated siRNAs per gene, and we confirmed that all of them showed inhibitory effects over *T. cruzi* infection with at least 2 of the 4 single siRNAs. The fact that even individual siRNA sequences were able to silence each gene, consequently decreasing infection of the host cells, allowed us to infer that those previous results were not under the influence of off-target effects, but rather were real hits.

We further checked if the hits found in the U2OS screening were also involved in the infection of HeLa cells, a cell line widely used for *T. cruzi* invasion studies. Our results demonstrated that most of the hits discovered in U2OS were also found to disturb *T. cruzi* infection in HeLa cells, pointing towards the existence of common pathways used by the parasite when infecting these different cell lines. On the other hand, we discovered that two of the hits, LOC389895 (Hypothetical LOC389895) and MGC33951 (Chromosome 15 open reading frame 43), were specific to U2OS cells, what suggests that the parasite may also make use of cell specific factors during infection.

Analysis of the 13 genes whose knockdown affected the *T. cruzi* infection process in U2OS cells revealed that several of the hits were genes coding for cell membrane proteins, which may be involved in signaling cascades triggered by *T. cruzi* or in the attachment of these parasites to the host cells. The five hits that localize at the cellular membrane are calcium binding protein 2 (CABP2), cadherin 11 type 2 OB-cadherin (osteoblast) (CDH11), olfactory receptor family 2 subfamily T member 5 (LOC401993), protocadherin alpha 13 (PCDHA13), and proline-rich membrane anchor 1 (PRIMA1). Other hits have their location inferred by similarity to their protein family members, such as calcium binding protein P22 (CHP), which is located at the cell cytoplasm (UniProt accession number Q99653), fucosyltransferase 8 (FUT8), which is present at the Golgi membrane (UniProt accession number Q9BYC5), and chemokine C-C motif ligand 4-like 1 (CCL4L1) and collagen type VI alpha 6 (LOC131873), which are both secreted proteins (UniProt accession numbers Q8NHW4 and A6NMZ7, respectively). Functionally, four of the hits are related to calcium-ion binding, which is consistent with previous evidence that *T. cruzi* triggers Ca^2+^ release on host cells to initiate the invasion process [9], [42]–[45]. The hits C20orf200, FLJ32783, LOC389895, MGC33951 and NICE-3 did not provide any information regarding subcellular location or molecular function because they are hypothetical or uncharacterized proteins. Thus, further experiments are necessary to validate these potential targets.

Particularly interesting was the fact that, in our screening, we identified several genes related to the TGF-beta-1 receptor, including FUT8 and CDH11. TGF-beta receptors are associated with *T. cruzi* infection in mammalian cells because inhibition of TGF-beta signaling *in vivo* decreases infection and prevents heart damage in mice [46]. In addition, secreted trypomastigote molecules are able to stimulate TGF-beta receptors in epithelial cells; treatment with TGF-beta greatly enhances *T. cruzi* invasion, while cell lines that lack these receptors have been shown to be resistant to *T. cruzi* infection [47]. There is evidence that the FUT8 protein, one of our hits, can increase TGFBR1 activation in embryonic fibroblasts and that mouse Fut8-deficient cells exhibit marked dysregulation of the TGF-β1 receptor and intracellular signaling [48], [49]. In agreement with those previous findings, we show here that U2OS cells bearing low levels of FUT8 protein exhibit a decreased infection ratio, potentially due to inefficient TGF-beta pathway signaling.

CDH11, also known as cadherin 11, is another protein member of the TGF-beta signaling pathway that we identified in our screenings. Members of the TGF-beta superfamily indirectly modulate cell adhesion by controlling cell surface levels of cadherins, integral membrane proteins that mediate calcium-dependent cell-cell adhesion [50], [51]. Cell-cell adhesion molecules expressed by host cells have also been shown to act as receptors for the binding of infectious agents [52]–[54]. In *Listeria monocytogenes*, for example, Internalin A protein is essential for efficient bacterial penetration into human epithelial cells, and this property is mediated by its binding to human E-cadherin in a Ca^2+^-dependent manner [53]. Another example is the neural cell adhesion molecule [NCAM], considered one of the *in vivo* receptors for the rabies virus in mice [54]. This same NCAM is an important cell-cell adhesion protein found in cardiomyocytes and may also act as a receptor for tissue targeting and cellular invasion by *T. cruzi* in Chagas disease. This idea is supported by a previously published study that showed that these parasites expressed NCAM-like proteins and that cellular NCAM was reported to be upregulated in Chagas disease myocarditis [55]. N-cadherins are very abundant proteins in the mammalian heart as well, and although no evidence has been found identifying cadherins as receptors involved in *T. cruzi* entry into host cells, it is reasonable to hypothesize that they might play a role during the *T. cruzi* infection process, not only as potential receptors, but also as signaling mediators.

Other hits found in this screening that were categorized as secreted proteins, such as chemokine C-C motif ligand 4-like 1 (CCL4L1) and collagen type VI alpha 6 (LOC131873/COL6A6), could have direct implications on the course of infection. Whereas the former is a chemokine involved in immunoregulatory and inflammatory processes [56], the latter is an extracellular matrix component that helps cells remain attached to the matrix to maintain tissue integrity [57]. Specific interactions between *T. cruzi* molecules and components of the extracellular matrix have already been described in previous studies [58]–[60]. In addition, *T. cruzi* trypomastigotes present on their surface collagen-binding proteins that can be involved in cell-parasite interaction [60].

Previous work using RNAi has shown that genes like laminin γ-1 [17], thrombospondin-1 [18], and cytokeratin 18 [19] are important for *T. cruzi* invasion of smooth muscle or HeLa cells. The fact that we did not identify these genes in our screening may be explained by differences in specific assay conditions used in the various studies that can greatly influence the results of screening, including cell type, parasite strain and/or infection time.

In summary, using genome-wide RNAi, we identified 14 host cell genes that proved to be necessary for *T. cruzi* infection, exposing as yet unrecognized factors in the host-pathogen interplay. The analysis of these key players and the molecular basis of their interactions will enable us to better understand the pathogenesis of Chagas disease and potentially reveal new targets for antiparasitic therapies. In addition, we have demonstrated that the experimental approach employed in this study is a valid tool to screen for human host factors that play a role in *T. cruzi* infection, and it is applicable to the study of factors involved in the infection of other pathogenic organisms as well.

## Supporting Information

Figure S1 **Schematic representation of the assay design.** U2OS cells were seeded over 7 glass slides containing 3,888 spots each. The siRNA was reverse transfected into the cells, and 48 hours later the transfected cells were infected with *T. cruzi* trypomastigotes. After 8 hours, the free parasites were washed out and the slides were incubated for an additional 28 hours. Cells and parasites were then fixed in 4% paraformaldehyde and stained with DRAQ5 for imaging. All slides were imaged in two channels, one for the spots stained with siGLO Red and another for cells stained with DRAQ5.
(TIF)

Video S1 **Video showing the software analysis in operation.** This video shows a whole microarray slide being analyzed by our customized software. First, a plug-in for fitting the gene information over each spot is executed (pink squares), providing spot localization on the microarray slides and showing all the missing spots (green squares). When the arrow points to a particular spot, the gene name and spot coordinates are shown, providing specific information related to that gene. In the right panel, the analysis plug-in is running, showing individual cell detection and quantification in operation. At the end of the process, a high-content description of each experiment is obtained, including infection and normality measurements. The spots are pseudocolored in red. For more information, see Materials and Methods.
(MPG)

Table S1 **Primers used for qRT-PCR.**
(DOC)
