# Supplementary figures and images for: Visual Genome-Wide RNAi Screening to Identify Human Host Factors Required for Trypanosoma cruzi Infection
Source: PLoS One. 2011 May 20;6(5):e19733. doi: 10.1371/journal.pone.0019733 (PMC3098829; doi:10.1371/journal.pone.0019733)

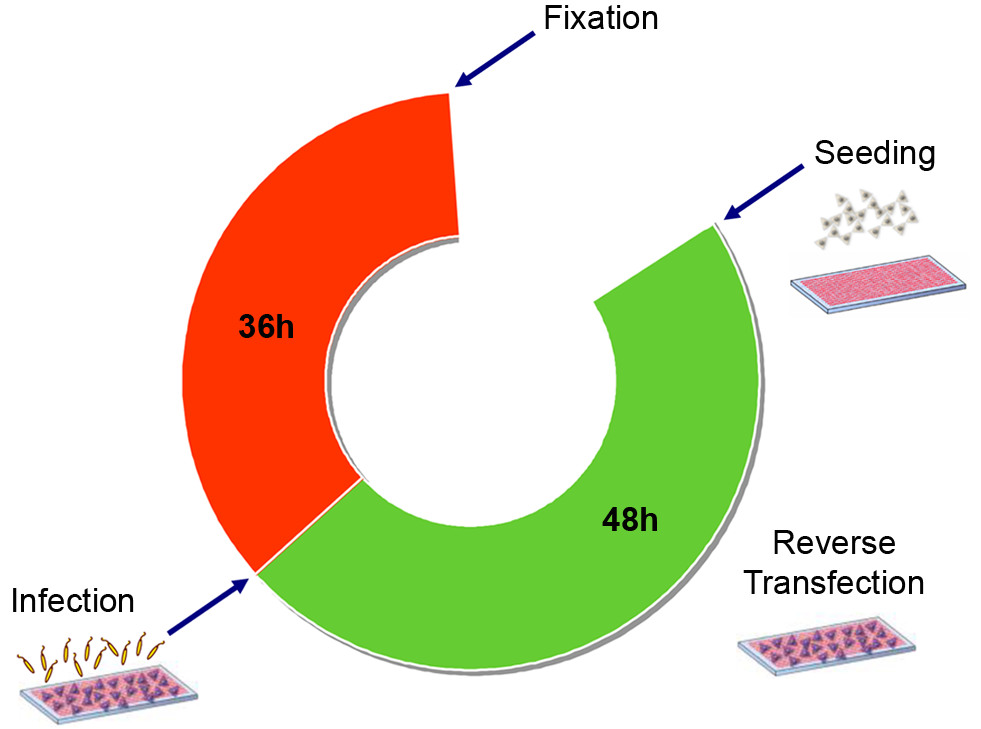

Supplement: Figure S1 — Schematic representation of the assay design. U2OS cells were seeded over 7 glass slides containing 3,888 spots each. The siRNA was reverse transfected into the cells, and 48 hours later the transfected cells were infected with T. cruzi trypomastigotes. After 8 hours, the free parasites were washed out and the slides were incubated for an additional 28 hours. Cells and parasites were then fixed in 4% paraformaldehyde and stained with DRAQ5 for imaging. All slides were imaged in two channels, one for the spots stained with siGLO Red and another for cells stained with DRAQ5. (TIF) [file pone.0019733.s001.tif]
