# Supplementary material for: Visual Genome-Wide RNAi Screening to Identify Human Host Factors Required for Trypanosoma cruzi Infection
Source: PLoS One. 2011 May 20;6(5):e19733. doi: 10.1371/journal.pone.0019733 (PMC3098829; doi:10.1371/journal.pone.0019733)
Supplement: Table S1 — Primers used for qRT-PCR. (DOC) [file pone.0019733.s003.doc]

**Table S1.** Primers used for qRT-PCR

| **Gene** | **Forward primers** | **Reverse primers** |
| --- | --- | --- |
| **CDH11** | 5’ - CGTGCTTGTGGGCAGGCTTCA - 3’ | 5’ - TGTCCACCGCCTGAGCCATCA - 3’ |
| **CHP** | 5’ - CGGGCCTCCACGTTACTGCG - 3’ | 5’ - TCTTCCCGGCTGAGAGTCCC - 3’ |
| **FUT8** | 5’ - TCATCCCAGGTCTGTCGAGTTGC - 3’ | 5’ - GTGGGCATTCTGGCCCCCAA - 3’ |
| **NICE-3** | 5’ - GAAGGCCGGCATCCCCGTTC - 3’ | 5’ - TGGCCAAAGACCCCTGTCCCA - 3’ |
